# Polyphasic Identification and Genomic Insights of *Leptothermofonsia sichuanensis* gen. sp. nov., a Novel Thermophilic Cyanobacteria Within Leptolyngbyaceae

**DOI:** 10.3389/fmicb.2022.765105

**Published:** 2022-03-28

**Authors:** Jie Tang, Mahfuzur R. Shah, Dan Yao, Ying Jiang, Lianming Du, Kelei Zhao, Liheng Li, Meijin Li, Michal M. Waleron, Malgorzata Waleron, Krzysztof Waleron, Maurycy Daroch

**Affiliations:** ^1^Antibiotics Research and Re-evaluation Key Laboratory of Sichuan Province, Sichuan Industrial Institute of Antibiotics, Chengdu University, Chengdu, China; ^2^School of Environment and Energy, Peking University Shenzhen Graduate School, Shenzhen, China; ^3^Department of Pharmaceutical Microbiology, Faculty of Pharmacy Medical University of Gdańsk, Gdańsk, Poland; ^4^Laboratory of Plant Protection and Biotechnology, Intercollegiate Faculty of Biotechnology University of Gdańsk and Medical University of Gdańsk, University of Gdańsk, Gdańsk, Poland

**Keywords:** 16S rRNA, 16S-23S ITS, genomics, thermophilic cyanobacterium, Leptolyngbyaceae, *Kovacikia*, *Stenomitos*

## Abstract

Thermal environments are an important reservoir of thermophiles with significant ecological and biotechnological potentials. However, thermophilic isolates remain largely unrecovered from their habitats and are rarely systematically identified. In this study, we characterized using polyphasic approaches a thermophilic strain, PKUAC-SCTAE412 (E412 hereafter), recovered from Lotus Lake hot spring based in Ganzi prefecture, China. The results of 16S rRNA/16S-23S ITS phylogenies, secondary structure, and morphology comparison strongly supported that strain E412 represent a novel genus within Leptolyngbyaceae. This delineation was further confirmed by genome-based analyses [phylogenomic inference, average nucleotide/amino-acid identity, and the percentages of conserved proteins (POCP)]. Based on the botanical code, the isolate is herein delineated as *Leptothermofonsia sichuanensis* gen. sp. nov, a genus adjacent to recently delineated *Kovacikia* and *Stenomitos*. In addition, we successfully obtained the first complete genome of this new genus. Genomic analysis revealed its adaptations to the adverse hot spring environment and extensive molecular components related to mobile genetic elements, photosynthesis, and nitrogen metabolism. Moreover, the strain was capable of modifying the composition of its light-harvesting apparatus depending on the wavelength and photoperiod, showing chromatic adaptation capacity characteristic for T1 and T2 pigmentation types. Other physiological studies showed the strain’s ability to utilize sodium bicarbonate and various sulfur compounds. The strain was also shown to be diazotrophic. Interestingly, 24.6% of annotated protein-coding genes in the E412 genome were identified as putatively acquired, hypothesizing that a large number of genes acquired through HGT might contribute to the genome expansion and habitat adaptation of those thermophilic strains. Most the HGT candidates (69.4%) were categorized as metabolic functions as suggested by the KEGG analysis. Overall, the complete genome of strain E412 provides the first insight into the genomic feature of the genus *Leptothermofonsia* and lays the foundation for future global ecogenomic and geogenomic studies.

## Introduction

Thermophilic cyanobacteria are photoautotrophic prokaryotes that inhabit inhospitable niches, such as hot springs and other thermal environments. These thermophiles are essentially primary producers of the geothermal ecosystems with substantial ecological importance (Esteves-Ferreira et al., 2018; Li et al., 2021). Besides, thermophilic cyanobacteria and their secondary metabolites have shown some potentials for biotechnological applications, including biosynthesis of thermostable metabolites and carbon valorisation vehicles (Patel et al., 2019). Unfortunately, thermophilic isolates remain largely unrecovered from their habitats to date and lack comprehensive knowledge about their physiology, ecology, systematics, and adaptations to the adverse thermal habitats.

Recently, next-generation sequencing (NGS) has been extensively applied to explore the cyanobacterial diversity in thermal environments (Tang et al., 2018b; Alcorta et al., 2020; Chen et al., 2021). However, NGS only generates ample abstract data and its assembly often results in unspecified operational taxonomic units (OTUs) and higher taxonomic groups. Isolation of thermophilic cyanobacteria from different ecosystems is fundamental for detailed characterization of their morphology, genetics, physiology, and biochemistry and for a better understanding of thermal ecology and their adaptation to thermal habitats (Cordeiro et al., 2020; Tang et al., 2022). However, the simple morphology of cultivated cyanobacteria makes these strains ambiguous and their taxonomic allocation challenging (Komárek and Anagnostidis, 2005). To address this problem, polyphasic taxonomic classification approaches have been widely demonstrated to be effective for cyanobacterial identification, especially for understudied or unresolved polyphyletic families/genera/species and identification of novel families and genera (Raabova et al., 2019; Shalygin et al., 2020). Consequently, establishing new genera as references will benefit the reallocation of numerous taxonomically ambiguous strains. For instance, *Leptolyngbya*-like strains have been scattered across phylogenies based on 16S rRNA or multi-locus sequence analysis within or even beyond family Leptolyngbyaceae (Sciuto and Moro, 2016; Yao et al., 2021). As the taxonomic delineation of isolates through systematic identification, numerous *Leptolyngbya*-like strains were assigned to new genus or species, such as *Allonema*, *Enugrolinea*, and *Thermoleptolyngbya* (Sciuto and Moro, 2016; Walter et al., 2017).

Genomic studies on thermophilic cyanobacteria are increasingly popular, and the better availability of complete genome sequences from thermal isolates and Metagenome-Assembled Genomes (MAGs) allows the studies of taxonomic relationships and genomic adaptations to different ecological niches among thermophilic strains and provides insight into survival strategies in extreme conditions not fully comprehended up to now (Chen et al., 2021). In addition, the acquisition of a complete genome may provide novel insights into the genomic features of new thermophilic microorganisms. More importantly, under the current scenario of global warming, it is important to understand the evolution of hot spring genomes as an example of selective pressure in warmer environments (Alcorta et al., 2020). Therefore, the acquisition of genomes from thermophilic cyanobacteria as much as possible is a prerequisite for such studies.

In the current study, we collected and analyzed morphological, physiological, and molecular data for the representative of an entirely novel genus of a filamentous thermophilic cyanobacterium isolated from a Lotus Lake hot spring based in Ganzi prefecture western Sichuan province of China. After thorough taxogenomic evaluation combined with 16S rRNA/16S-23S ITS phylogenies, secondary structure, and morphology comparison, a new name *Leptothermofonsia sichuanensis* gen. sp. nov. has been proposed based on the botanical code for the strain as the first representative of genus *Leptothermofonsia*. Furthermore, genomic features of this genus were studied, and some genes were correlated with the physiological properties.

## Materials and Methods

### *Leptothermofonsia sichuanensis* gen. sp. nov. E412: Origins, Cultivation, and Basic Physiological Assessment

The strain E412 presented in the current study was initially isolated from Lotus Lake hot springs in the Ganzi region of Sichuan Province, China. Information about the sampling site and preliminary taxonomic allocation of the strain was reported in our previous studies (Tang et al., 2018a,b). The unicyanobacterial culture of the strain was cryopreserved as 10% DMSO in BG11 stocks in −80°C. Final precultures for experiments were established as previously described (Tang et al., 2018b) and cultivated at 45°C in 150 mL BG-11 medium in 500 mL Erlenmeyer flasks agitated at 100 rpm under 12L:12D photoperiod at 45 μmol m^–2^ s^–1^ provided by fluorescent tubes unless stated otherwise. The strain initially denoted as PKUAC-SCTE412 has been recently deposited in the Freshwater Algae Culture Collection at the Institute of Hydrobiology (FACHB-collection) with an accession number FACHB-2490. Physiological assessment of the strain was performed using BG-11 medium free of an essential nutrient (nitrogen or sulfur) supplemented with 17 mM NaNO_2_, 85 mM NaNO_3_, 10 mM Na_2_SO_4_, and 10 mM NaHSO_3_. The nitrogen fixation capacity during 72 h was performed using the methodology described by Li et al. (2021).

The chromatic adaptation capacity of strain E412 was assessed by culturing the cells at constant LED illumination using either white light (6,500 K) or far-red light (730 nm) at the intensity of 250 and 25 μmol m^–2^ s^–1^, respectively. After 15 days, cyanobacterial cells were collected to measure chlorophyll a, chlorophyll b, carotenoids, allophycocyanin, phycocyanin, and phycoerythrin, according to previously published protocols (Bennett and Bogorad, 1973; Dere et al., 1998). Lipophilic and water-soluble pigments from 15 mg of lyophilized biomass were extracted with 100% methanol and phosphate-buffered saline (PBS), respectively. The absorbance of the supernatant at 470, 562, 615, 652, 653, and 666 nm were recorded against the relative blank using a UV-Vis spectrophotometer (Shimadzu UV-1,800, Japan). The concentration of pigments was calculated using respective formulae for chlorophylls and carotenoids (Dere et al., 1998) and phycobiliproteins (Bennett and Bogorad, 1973).

### Genome Sequencing, Assembly, and Annotation of Strain E412

Genomic DNA was extracted and assessed following the previously described method (Tang et al., 2018a). The whole genome of E412 was sequenced using a combination of Oxford Nanopore Technologies (ONT) and Illumina short-read approaches. Illumina sequencing of E412 genome generated 6,810,074 filtered paired-end reads (clean data), providing approximately 185-fold coverage of the genome. The clean data were assembled into contigs using MaSuRCA v. 3.4.1 with default parameters (Zimin et al., 2013) and Flye for final assembly, generating a single contig after manual curation. The draft circular genome was error-corrected with Illumina NovaSeq reads using Burrows-Wheeler Aligner, BWA v0.7.17 (Li and Durbin, 2009), and then Pilon v1.23 (Walker et al., 2014). The complete genome has been deposited in GenBank with an accession number CP072600.

The annotation of the E412 genome was performed using a pipeline described by Tang et al. (2019). In short, initial gene prediction and annotation were completed using the automatic NCBI prokaryotic genome annotation pipeline (Tatiana et al., 2016). Subsequently, poor calls were corrected using the RAST annotation system. The insertion sequence (IS) was detected and annotated by ISsaga (Varani et al., 2011). Prophage regions and CRISPR/Cas loci were detected by PHASTER (David et al., 2016) and by CRISPRCasFinder server (Couvin et al., 2018), respectively.

For functional classification, the predicted protein sequences were searched against the NCBI non-redundant database using BLASTP with an *E*-value cutoff of 1e-5. The search outputs were imported into BLAST2GO V5.2.5 (Conesa et al., 2005) for GO term mapping. GO classification was subsequently conducted by WEGO (Ye et al., 2006) using the following ontologies: biological process, molecular function, and cellular component. Additionally, KEGG orthology (KO) identifiers were assigned to predicted protein sequences by KofamKOALA (Aramaki et al., 2019), and then BRITE mapping process was performed online.^1^

### Phylogenetic Reconstruction of 16S rRNA and 16S-23S ITS

The 16S rRNA gene and 16S-23S ITS regions were extracted from the E412 for phylogenetic analysis. Cyanobacterial sequences were retrieved from GenBank through BLAST search as references to construct datasets for phylogenetic analyses of 16S rRNA gene and 16S-23S ITS, respectively. Multiple alignments of sequences were performed by Muscle implemented in Mega7 (Kumar et al., 2016) and manually edited where necessary. Maximum-Likelihood (ML) phylogenetic analyses were carried out using PhyML v3.0 (Guindon et al., 2010), and the substitution models were selected by the Model Selection function implemented in PhyML (Vincent et al., 2017) under Akaike information criterion (AIC). A non-parametric bootstrap test (1,000 replications) was performed to assess the robustness of tree topologies.

### Prediction of Secondary Structures

The conserved domains of the 16S-23S ITS region: D1-D1′, D2, D3, boxA, and D4; and its variable regions (V2, boxB, and V3) were identified as previously described (Iteman et al., 2000). The tRNAs presented in the spacer were identified by tRNAscan-SE v1.3.1 (Lowe and Eddy, 1997). The secondary structures of the identified fragments were individually determined by RNAstructure web server using default settings (Mathews, 2014).

### Microscopic Analysis

Strain E412 was investigated at 400 × magnification using light microscopy (LM, DP72, OLYMPUS, Japan), equipped with an image acquisition system (U-TV0.63XC, OLYMPUS, Japan). Microscopic observations were also performed using scanning electron microscopy (SEM) (SU8100, HITACHI, Japan), and using transmission electron microscopy (TEM) (HT7800, HITACHI, Japan), essentially as described before (Tang et al., 2022).

### Genome-Based Analysis

To compare divergence in genomes of focal taxa (E412 and previously established genera within Leptolyngbyaceae), a high-quality dataset comprising genomes with near completeness (≥ 90%) and low contamination (<5%) was constructed to calculate whole-genome average nucleotide identity (ANI) and average amino acid identity (AAI) using the ANI/AAI calculator with default settings.^2^ In addition, the percentages of conserved proteins (POCP) between the E412 genome and focal taxa were pairwise calculated to estimate their evolutionary and phenotypic distance. Finally, the POCP was determined for prokaryotic genus delineation according to the method described previously (Qin et al., 2014).

The concatenated sequences from single-copy genes shared by all the genomes were used to elucidate the phylogenomic relationship between E412 and focal taxa. Single-copy genes shared by all genomes were refined from the homologous gene clusters identified by OrthoMCL (Li et al., 2003) and concatenated using custom Perl script. MAFFT v7.453 (Standley, 2013) was used to generate multisequence alignment. The supergene alignment was subjected to phylogenomic inference using IQ-TREE v2.1.3 (Minh et al., 2020). ModelFinder implemented in IQ-TREE was employed to select the optimal substitution model for phylogenomic analysis from 546 protein models. Bootstrap tests (1,000 replicates) were performed to assess tree topologies using UltraFast Bootstrap (Hoang et al., 2018). A strain from the family Oculatellaceae, *Thermoleptolyngbya sichuanensis*, was rooted as an outgroup.

### Taxonomic Evaluation

For a valid description, the classification system applied was based on Komárek et al. (2014), and the taxon description follows the prescriptions of the Botanical Code, International Code of Nomenclature for Algae, Fungi, and Plants (Shenzhen code) (Turland et al., 2018).

### Identification of Horizontal Gene Transfer Candidates

A BLASTP-based approach was employed to identify potential genes acquired through horizontal gene transfer (HGT). BLASTP searches were performed against the NCBI non-redundant protein database (last accessed January 20, 2018). The pairs that accounted for at least 90% of the query length and amino acid sequence similarity of at least 40% were used for the HGT detection. Taxonomic classification was assigned to each hit with the taxonomy files downloaded from the NCBI database. Protein sequences were identified as HGT candidates if all the top-five hits were not from the family Leptolyngbyaceae.

## Results and Discussion

### Phylogeny of 16S rRNA Gene

**FIGURE 1:**
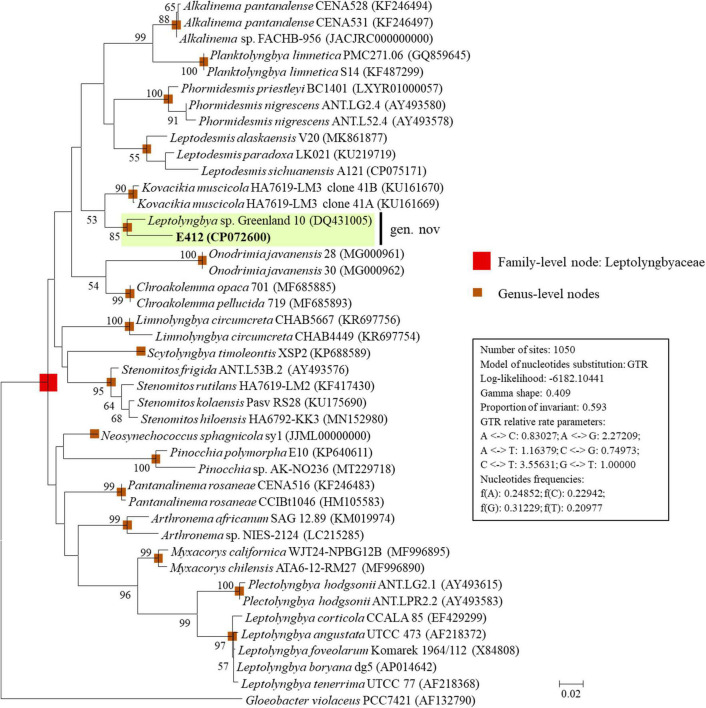
ML phylogenetic tree of 16S rRNA gene sequences. Strain no. in bold represent the strains identified in this study. Only bootstrap values > 50% (1,000 non-parametric replications) are indicated at nodes. Scale bar = 2% substitutions per site.

The ML phylogram (Figure 1) inferred from 16S rRNA gene sequences recognized 17 well-supported clades of previously established genera: *Alkalinema* (Vaz et al., 2015), *Arthronema* (Komárek and Lukavský, 1988), *Chroakolemma* (Becerra-Absaln et al., 2018), *Kovacikia* (Miscoe et al., 2016), *Leptodesmis* (Raabova et al., 2019), *Leptolyngbya sensu stricto* (Taton et al., 2010), *Limnolyngbya* (Li and Li, 2016), *Myxacorys* (Soares et al., 2019), *Neosynechococcus* (Dvorak et al., 2014), *Onodrimia* (Jahodářová et al., 2017), *Pantanalinema* (Vaz et al., 2015), *Phormidesmis* (Raabova et al., 2019), *Pinocchia* (Dvorak et al., 2015), *Planktolyngbya* (Thomazeau et al., 2010), *Plectolyngbya* (Taton et al., 2011), *Scytolyngbya* (Song et al., 2015), *Stenomitos* (Miscoe et al., 2016), and *Gloeobacter* as an outgroup of the phylogram. Strain E412, together with *Leptolyngbya* sp. Greenland 10, formed a well-defined clade that was phylogenetically novel to the other described taxa (Figure 1), suggesting a new genus within the family Leptolyngbyaceae.

The sequence identity (Supplementary Table 1) indicated that the nearest neighbor of E412 was *Leptolyngbya* sp. Greenland 10 (96.2% identity), a poorly described strain isolated from the hot spring (56–61°C), Rømerfjord, Greenland (Roeselers et al., 2007), followed by *Kovacikia* (94.9–95.0% identity) and *Pantanalinema* (94.0% identity). The other focal taxa exhibited 89.3–93.9% identities of 16S rRNA gene to strain E412 (Supplementary Table 1). According to the recommended threshold of 16S rRNA gene identity for bacterial species (98–99%) or genera (94.5–95%) demarcation (Rodriguez-R et al., 2018), strain E412 was proposed to be categorized into a new genus within the family Leptolyngbyaceae. And *Leptolyngbya* sp. Greenland 10 was also assigned to this new genus but a different species based on the 16S rRNA gene identity. This was also supported by the ML topology of the 16S rRNA gene that Greenland 10 formed a distinct sister branch alongside strain E412 (Figure 1). Interestingly, the two strains within the proposed genus were both isolated from thermal habitats, but the isolation sources were geographically divergent and exhibited distinct geochemical characteristics (Roeselers et al., 2007; Tang et al., 2018a). Notably, the newly established genus offers a solid basis for further investigations or comparisons on numerous aspects, such as whether the proposed genus has a cosmopolitan distribution and all the strains within this genus are specific to thermal habitat.

### Phylogeny of 16S-23S ITS

**FIGURE 2:**
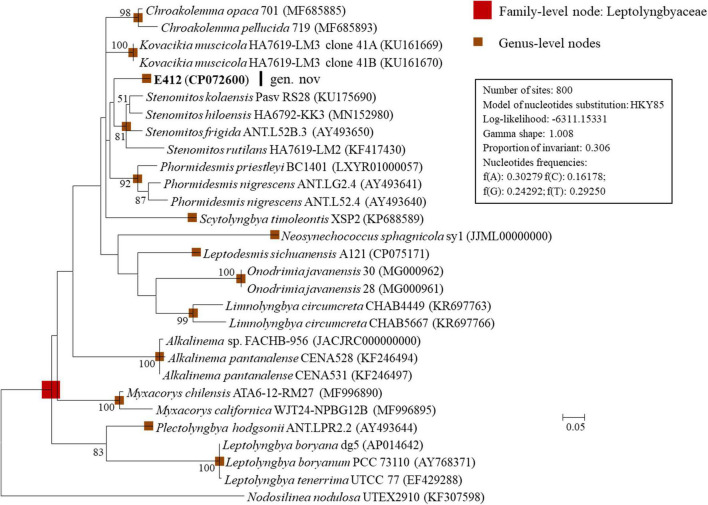
ML phylogenetic tree of 16S-23S ITS sequences. Strain no. in bold represent the strains identified in this study. Only bootstrap values > 50% (1,000 non-parametric replications) are indicated at nodes. Scale bar = 5% substitutions per site.

Additional to the 16S rRNA gene, the 16S-23S ITS region has also been commonly recommended to establish cyanobacterial ecotypes or species (Brito et al., 2017). Several genera included in the 16S rRNA phylogram were excluded in the ITS phylogenetic analysis due to sequence unavailability. As shown in Figure 2, strain E412 was placed into a well-separated branch in the ITS phylogram. Previously described genera were also recognized in the 16S-23S ITS phylogram and supported by robust bootstrap values. However, the ITS phylogram (Figure 2) showed inconsistent topology to that of the 16S rRNA gene (Figure 1). Previous reports have manifested that the sequences of 16S-23S ITS were extraordinarily divergent (Johansen et al., 2011; Tang et al., 2021). Furthermore, regions/domains within ITS were occasionally absent in some cyanobacterial strains, e.g., in the present study, conserved tRNAs were absent in *Pantanalinema rosaneae* (Vaz et al., 2015). Taken together, phylogenetic inference of ITS could result in erroneous taxonomic classification. Therefore, secondary structure analyses of domains in 16S-23S ITS were also carried out as an essential complement to the final taxonomy determination.

### 16S-23S ITS Secondary Structures

Strain E412 and representative strains of 13 focal genera within the family Leptolyngbyaceae were used for 16S-23S ITS secondary structure analysis. After removing the two highly conserved tRNAs (Ile and Ala) from ITS sequences, the remaining ITS sequences varied in length from 230 to 388 bp (Table 1). Such variation was primarily ascribed to the length differences in D1-D1′ (51–121 bp), V2 (7–90 bp), boxB (33–63 bp), and V3 (19–98 bp (Table 1). Consequently, the nucleotide differences of these domains among strains resulted in divergences of the secondary structures.

**TABLE 1:** The length (bp) summary of regions within16S-23S ITS of Leptolyngbyaceae strains studied.

Strain	ITS length (tRNA removed)	D1-D1′ helix	D2	D3	tRNA^Ile^	boxA	D4	V2 helix	tRNA^Ala^	boxB helix	V3 helix
E412	380	121	12	5	74	12	7	76	73	45	98
*Alkalinema pantanalense* CENA528	296	64	12	5	74	12	7	24	73	48	54
*Chroakolemma pellucida* 719	268	61	12	5	74	12	7	16	73	41	53
*Kovacikia muscicola* HA7619-LM3 clone 41A	345	63	12	5	74	12	7	90	73	41	95
*Leptodesmis sichuanensis* A121	325	63	12	5	74	12	7	81	73	33	98
*Leptolyngbya boryanum* PCC 73110	275	51	12	5	74	12	7	10	73	33	21
*Limnolyngbya circumcreta* CHAB5667	388	98	12	5	74	12	7	83	73	63	76
*Myxacorys californica* WJT24-NPBG12B	258	86	12	5	74	12	7	9	73	33	71
*Neosynechococcus sphagnicola* sy1	230	63	12	5	74	12	7	11	73	39	95
*Onodrimia javanensis* 28	280	105	12	5	74	12	7	7	73	44	47
*Phormidesmis priestleyi* ANT.L52.4	329	113	12	5	74	12	7	12	73	56	77
*Plectolyngbya hodgsonii* ANT.LPR2.2	306	55	12	5	74	12	7	29	73	44	19
*Scytolyngbya timoleontis* XSP2	276	64	12	5	74	12	7	14	73	40	94
*Stenomitos rutilans* HA7619-LM2	258	65	12	5	74	12	7	7	73	34	92

The inferred D1-D1′ helix of strain E412 (Supplementary Figure 1) was distinct from the other inferred structures. The most similar structure to D1-D1′ helix of strain E412 was that of *O. javanensis*. Both D1-D1′ helix of the two strains were mainly composed of a stem fragmented by asymmetrical/symmetrical loop, right/left bulge, and terminated with hairpin loop. But the structures of the two strains varied in the residue size and/or the number of loops, bulges, and terminal hairpins. In addition, the fragmented stems also differed in number and length between the two strains.

The hypothetical V2 helix of strain E412 (Supplementary Figure 2) was divergent from the other analyzed strains. The tremendous length variations were responsible for the divergence in secondary structures. The V2 helix of strain E412 was straightforward and comprised 5 stems, 4 symmetrical loops, and an 8-residue hairpin loop. Although *K. muscicola*, *Leptodesmis* sp., and *L. circumcreta* possessed similar sequence lengths with respect to the strain E412, the helices differed in the stem, loop, bulge, and hairpin.

A basal stem structure (AGCA-UGCU) was shared by boxB helices of all strains except for *A. pantanalense* (Supplementary Figure 3). Strain E412 showed similar residue length to several strains, but no boxB helix structures were analogous to that of strain E412. The boxB helix of strain E412 was mainly composed of a stem orderly fragmented by single base right bulge, single base left bulge, 10-residue symmetrical loop, and terminated with a 5-residue hairpin loop.

Strain E412 exhibited the longest V3 helix (98 residues) (Table 1), comprising 3 asymmetrical loops, 3 symmetrical loops, a single base left bulge, an 8-residue hairpin loop, and fragmented stems (Supplementary Figure 4). The V3 helix of strain E412 was distinct from those of the other strains, whereas a basal stem structure (GUC-GAC) was shared by all the strains. Although the helix length of *K. muscicola* (95 residues), *N. sphagnicola* (95 residues), *S. timoleontis* (94 residues), and *S. rutilans* (92 residues) was similar to that of strain E412, the structures differed from each other in terms of bulge, loop, hairpin, and stem.

Conclusively, the result of 16S-23S ITS secondary structure analysis and the phylogenetic inferences of 16S rRNA and 16S-23S ITS verified strain E412 as a new genus within the family Leptolyngbyaceae. Our results (Supplementary Figures 1–4) also demonstrated that the secondary structure analysis of D1-D1′, V2, boxB, and V3 helix was an effective tool for genus-level identification within the family Leptolyngbyaceae.

### Genome-Based Analyses

**FIGURE 3:**
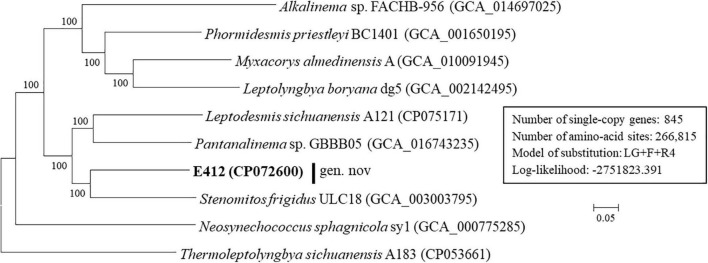
ML phylogenomic tree of concatenated protein alignment of single-copy genes shared by all genomes. Strain no. in bold represent the strain identified in this study. Bootstrap values (1,000 replications) are indicated at nodes. Scale bar = 5% substitutions per site.

Based on the genomes available on a public database, comparison analyses at the genomic level were performed among genera within Leptolyngbyaceae. Considerable divergences in genomes were observed among different genera as revealed by the ANI and AAI values (Table 2). Particularly, the ANI and AAI values between strain E412 and the other eight focal taxa were less than 79 and 69%, respectively. The results of genome-wide ANI and AAI conformed to the suggested values for genus (ANI < 83%, AAI ≤ 70%) delimitation (Walter et al., 2017; Jain and Rodriguez, 2018), further confirming the taxonomy delineation of a novel genus within the family Leptolyngbyaceae. However, it was reported that the classification of the prokaryotic genus using ANI or AAI might cause misleading results in some cases (Konstantinidis and Tiedje, 2005; Pannekoek et al., 2016). Therefore, the POCP specific for genus delineation was calculated to further verify the genus demarcation of strain E412. The POCP values (Table 3) between the E412 genome and focal taxa ranged from 35.7 to 49.3%, all within the threshold (<50%) for the definition of a prokaryotic genus (Qin et al., 2014). Additionally, the ML genomic phylogram (Figure 3) generated from the concatenated alignment of 845 single-copy genes showed a consistent topology to that of 16S rRNA, and again verified the conclusion that strain E412 belonged to a novel genus within Leptolyngbyaceae. Unfortunately, only some of the genera related to E412 had their genomes sequenced, resulting in a partial snapshot of genomic divergences among these organisms.

**TABLE 2:** Values of ANI (Average Nucleotide Identity) and AAI (Average Amino acid Identity) among Leptolyngbyaceae genomes studied.

Strain	E412	FACHB-956	A121	dg5	A	sy1	GBBB05	BC1401	ULC18
E412	*100.00*	60.87	68.49	62.45	63.54	61.83	67.45	63.93	67.86
*Alkalinema* sp. FACHB-956	75.64	*100.00*	60.97	61.62	62.35	57.98	60.93	62.31	59.56
*Leptodesmis sichuanensis* A121	78.79	75.72	*100.00*	62.61	63.55	62.09	68.21	64.29	66.56
*Leptolyngbya boryana* dg5	77.29	77.65	77.02	*100.00*	68.15	59.06	62.46	66.71	61.70
*Myxacorys almedinensis* A	74.40	78.58	72.91	74.88	*100.00*	59.58	63.12	67.93	62.91
*Neosynechococcus sphagnicola* sy1	75.81	75.63	76.83	76.60	71.80	*100.00*	61.92	60.79	62.14
*Pantanalinema* sp. GBBB05	74.40	74.49	75.41	77.67	74.84	73.88	*100.00*	64.33	66.26
*Phormidesmis priestleyi* BC1401	75.41	74.34	75.42	75.05	74.46	76.76	74.53	*100.00*	65.20
*Stenomitos frigidus* ULC18	74.46	73.53	73.79	74.59	73.60	75.81	74.91	77.97	*100.00*

*The numbers above and below the diagonal indicate the AAI and ANI values (%), respectively.*

**TABLE 3:** POCP values between strain E412 and representative species from Leptolyngbyaceae genera.

Species	E412
*Alkalinema* sp. FACHB-956	43.8%
*Leptodesmis sichuanensis* A121	48.5%
*Leptolyngbya boryana* dg5	44.6%
*Myxacorys almedinensis* A	45.2%
*Neosynechococcus sphagnicola* sy1	35.7%
*Pantanalinema* sp. GBBB05	49.3%
*Phormidesmis priestleyi* BC1401	44.1%
*Stenomitos frigidus* ULC18	47.6%

### Morphological Characteristics of Strain E412

**FIGURE 4:**
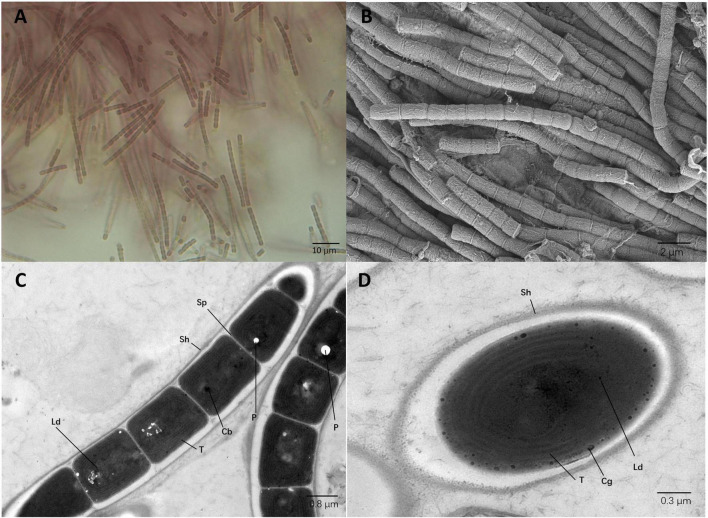
Micrographs of strain E412. **(A)** Light microscopy image. **(B)** SEM image. **(C,D)** TEM images. Cb, carboxysome; Cg, cyanophycin granule; Ld, lipid droplet; P, polyphosphate body; Sh, sheath; Sp, septum; T, thylakoid membrane. Magnifications were 1,000× **(A)**, 5,000× **(B)**, 8,000× **(C)**, and 12,000× **(D)**.

Analysis of strain E412 with light microscopy revealed that trichomes were brown and exhibited coiled and tangled morphology (Figure 4A). The SEM and TEM analysis indicated unbranched trichomes composed of elongated cylindrical shaped cells, 1.2–1.8 μm in length and 0.8–1.1 μm in width. Constrictions were detected at the cross-walls of cells (Figures 4B,C). Centripetal invaginations of the cell wall separated individual cells of the filaments, but the intracellular connections between individual vegetative cells were not present (Figure 4C). The analysis of TEM micrographs indicated that five to six thylakoids in the parietal arrangement were present at the inner periphery of cells. Additionally, typical components of filamentous cyanobacteria, i.e., sheath, septum, phycobilisomes, and carboxysomes, were identified. The strain was also identified as a cyanophycin producer, and granules of this biopolymer were observed in the cytoplasm. Polyphosphate bodies and small lipid droplets were also identified (Figures 4C,D). Morphological comparison of strain E412 against other focus taxa from the family Leptolyngbyaceae (Table 4) revealed the strain’s closest morphological resemblance to *Stenomitos* and *Kovacikia*, in agreement with the phylogenetic allocation. All the three strains had single unbranched filaments, often entangled. Individual cells were approximately 1 μm in width, cylindrical, and slightly elongated. The cells of *Leptothermofonsia* and *Kovacikia* were typically not longer than 1.8 μm, as opposed to longer cells of *Stenomitos*. All the three strains were brown in the natural environment. Strain E412 can adapt to the green phenotype with the modification of light intensity and photoperiod. No such information was available for other strains. Among other focus taxa, only *Leptodesmis* was distinctly green and shown to be incapable of chromatic adaptation. Available morphological characteristics of focus taxa was collected and summarized in Table 4. Although strain E412 was also morphologically similar to *Pseudanabaena* spp. (Komárek and Anagnostidis, 2005), they were phylogenetically divergent from each other (Supplementary Figure 5).

**TABLE 4:** Comparison of morphological features of Leptolyngbyaceae strains.

Species	Morphology	Cell width (μm)	Cell length (μm)	Sheaths	Thylakoids No.	Color	References
E412	Coiled, tangled, closely packed	0.8–1.1	1.2–1.8	Colorless	5–6	Brown or green depending on conditions	This study
*Alkalinema pantanalense* CENA528	Entangled, flexuous	1.7–2.2	2.0–4.1	-	NA	Reddish or brownish	Vaz et al., 2015
*Kovacikia muscicola* HA7619-LM3 clone 41A	Straight, unbranched	1.0–1.4	1.0–1.4	Thin, colorless	NA	Brownish	Miscoe et al., 2016
*Leptodesmis paradoxa* LK021	Straight, curved, flexuous or wavy, solitary	2.5–3.5	1.0–1.5	Thick, colorless	NA	Pale blue-green	Raabova et al., 2019
*Phormidesmis nigrescens* LK013	Straight, curved, solitary	1.5–2.50	1.0–2.0	Thick, blackish	+	Blackish	Raabova et al., 2019
*Stenomitos rutilans* HA7619-LM2	Bent, entangled	0.9–1.1	2.5–5.0	-	NA	Red brownish	Miscoe et al., 2016

*NA, not available; +/−, presence/absence.*

### Physiological Characteristics of Strain E412

Strain E412 was physiologically characterized using different variants of the standard growth medium, BG-11. The strain exhibited active growth when sodium bicarbonate at the concentration ranging from 0.1 to 0.5 M was applied, suggesting that this form of inorganic carbon can be effectively utilized by the strain. Analysis of the impact of sulfur compounds showed that the strain was capable of utilizing 10 mM sulfates and incapable of utilizing equivalent concentrations of sulphites. The strain exhibited flexibility in the utilization of nitrogen sources. Both nitrate (3 mM) and nitrite (6 mM) resulted in the active growth of the strain. Strain E412 was also diazotrophic. The acetylene reduction assay indicated a functional nitrogenase capable of a steady increase of ethylene production during 72 h of the assay (Supplementary Figure 6).

**FIGURE 5:**
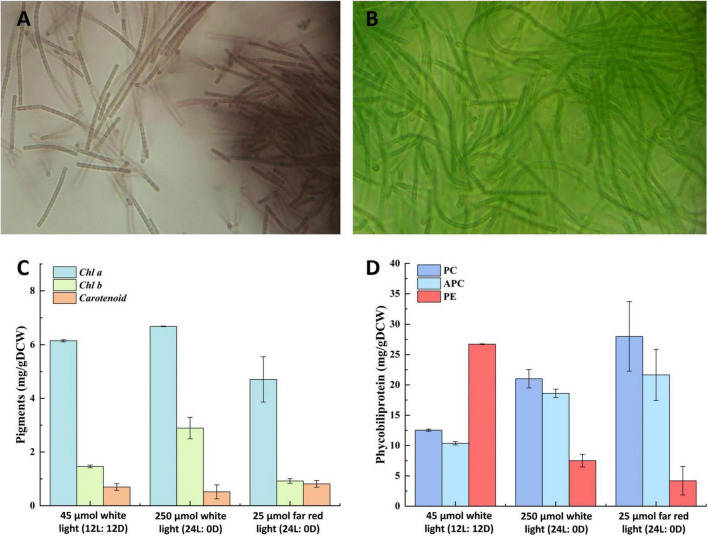
Chromatic adaptation of strain E412. **(A)** Light microscopy image (1,000×) of strain E412 grown in white fluorescent light, 12L: 12D photoperiod at 45 μmol m^–2^ s^–1^, **(B)** light microscopy image (1,000×) of strain E412 grown in far-red (730 nm) LED light, 24L: 0D photoperiod at 25 μmol m^–2^ s^–1^, **(C)** composition of photosynthetic lipophilic pigments of strain E412 grown at different illumination conditions, the figure represents a mean of three biological replicates, **(D)** composition of photosynthetic water-soluble pigments of strain E412 grown at different illumination conditions, the figure represents a mean of three biological replicates.

Analysis of the pigment composition of strain E412 under different illumination conditions suggested a significant difference in their composition at white light and far-red light illumination and across the different photoperiods (Figure 5). The strain cultivated at 12L:12D photoperiod in low light exhibited a brown phenotype and pigmentation typical of type T2 (Six et al., 2007) due to increased phycoerythrin accumulation (Figures 5A,D). Meanwhile, cultivation in high light and far-red light under constant illumination resulted in the recomposition of the photosynthetic apparatus and pigmentation type T1 (Figures 5B,D). Therefore, the strain can adjust its photosynthetic apparatus through chromatic adaptation and was capable of far-red light (730 nm) utilization like other thermophilic filamentous strains (Gan and Bryant, 2015). Meanwhile, the concentration of chlorophyll b increased in the cells grown in constant white light illumination (Figure 5C), whereas all the other pigments were relatively constant across the tested cultivation conditions (Figures 5C,D).

### General Genomic Features of Strain E412

The combined utilization of ONT and Illumina sequencing systems resulted in the complete genome of strain E412. This genome consisted of a single circular chromosome with a size of 6,426,061 bp (GC content, 50.8%). Two ribosomal RNA (*rrn*) operons, 74 tRNA genes and 7,904 protein-coding sequences (CDS), were predicted in the E412 chromosome (Supplementary Table 2). Notably, 4,015 out of 7,904 (50.8%) protein-coding genes were annotated as hypothetical proteins. Identifying such a high percentage of hypothetical protein in the E412 genome was not surprising and common to the genomes of thermophilic cyanobacteria (Cheng et al., 2020; Tang et al., 2021, 2022).

The GO analysis indicated that the CDS identified in the E412 genome were assigned to a wide range of functional categories (Supplementary Figure 7). The majority of GO terms were assigned to the biological process. Membrane, catalytic activity, and metabolic process were the most abundant GO term for cellular component, molecular function, and biological process, respectively. The most abundant GO terms distribution pattern was also noticed in another thermophilic strain, *T. sichuanensis* A183 isolated from a hot spring of Ganzi prefecture, China (Tang et al., 2021). However, the GO terms of the A183 genome were mostly concentrated only in eight functional categories. Meanwhile, their distribution in E412 was across many categories. Further KEGG pathway analysis showed that most genes (66.2%) in the E412 genome were distributed in the sub-category of metabolism (Supplementary Figure 8), suggesting that these metabolism-related genes may be crucial for this strain to survive in the oligotrophic aquatic environments of its origin.

### Horizontal Gene Transfer

Based on a stringent screening of BLASP results, 1,944 out of 7,904 (24.6%) annotated protein-coding genes were identified as putatively acquired (Supplementary Table 3). Many putatively acquired genes were also notable in other thermophilic cyanobacteria, e.g., *Thermosynechococcus* sp. CL-1 (19%) (Cheng et al., 2020). The putatively acquired genes through HGT may contribute to the genome expansion and acquisition of new functions and conceivably acclimation to variable environments of these thermophilic strains. For instance, 22 genes annotated as heat shock proteins were identified as putatively acquired (Supplementary Table 3), which might play a crucial role in adapting to the thermal environment. The putatively acquired gene, flavodoxin gene (*fldA*), may substitute the function of ferredoxin in the photosynthetic electron transport chain under iron-deficient conditions (Cao et al., 2020). Twelve out of 29 genes annotated as circadian input kinase A (*cikA*) were identified as acquired genes from diverse donors (Supplementary Table 3). Among the acquired *cikA* genes, three showed very low protein similarities (38.4–40.5%) to the kinase of *Synechococcus elongatus* PCC 7942, which was experimentally demonstrated as a critical factor for entraining the clock in the cyanobacterium (Ivleva et al., 2006). The distinct proteins of acquired *cikA* genes in the E412 genome implied that alternative circadian rhythms could be used to time metabolic and behavioral events with the external environment through entrainment. Concerning function, the HGT candidates in the E412 genome were assigned to a wide range of functional categories as revealed by both GO and KEGG analysis (Supplementary Figures 7, 8). The most represented GO term was catalytic activity, followed by metabolic process and cellular process. Interestingly, the majority of HGT candidates (69.4%) suggested by the KEGG analysis were categorized as being related to metabolic functions. This result was consistent with the complexity hypothesis that fundamental genes are less likely than peripheral and operational genes to be horizontally transferred (Thomas and Nielsen, 2005).

We noticed that the criteria for HGT detection were distinct among reported studies. For example, genus-level (Cheng et al., 2020) or cyanobacteria-level (Chen et al., 2021) were separately customized to identify acquired genes in light of research objectives. Although modified approaches have been employed in this study or other studies, taxon-sampling biases may exist in the high-throughput BLASP-based approach. In addition, data availability was also an important factor affecting the establishment of the analysis pipeline, since only one genome sequence was obtained for this genus. More genome sequences of this genus are required for further HGT analysis, such as identification of recently acquired genes.

### Mobile Genetic Elements

A total of 431 ISs (insertion sequences) corresponding to 67 different ISs were identified in the E412 genome. The IS630 family (67.29%) was dominant among the observed IS families, followed by the IS1 family (9.74%) and the IS4 family (7.66%). Besides, genes encoding transposase (86) were also noticed in abundance (Supplementary Table 2), indicating that intragenomic rearrangements might contribute much to the genetic plasticity of the strain.

One incomplete prophage loci was predicted in the chromosome, phiE412 (9.6 kb; positions 1,993,638–2,003,299). Thirteen phage-related genes were identified in the phiE412 region (Supplementary Table 4). No genes corresponding to DNA synthesis were found in the prophage loci, suggesting that this region was replication-defective. Repeats that constituted the core regions of phage attachment were not found to flank phiE412. Thus, this partial prophage loci probably was not functional. Clustered regularly interspaced short palindromic repeats (CRISPRs) were reported to function in the interference pathway to maintain genome integrity (Gasiunas et al., 2014). In the E412 chromosome, we detected three CRISPRs with high evidence levels and three Cas clusters assigned to type IA and III-D (Supplementary Table 5). However, the E412 genome had only one CRISPR-Cas array (type III-D), which was consistent with the finding in thermophilic strain *T. sichuanensis* A183 (Tang et al., 2021). Besides, several other genes typically associated with this system, e.g., *cmr*2, were also found to flank the array. The results indicated that this CRISPR-Cas interference system might function to limit HGT (Marraffini and Sontheimer, 2008).

### Thermotolerance

As a microorganism living in a hot spring (temperature: 67.2^°^C), strain E412 must possess survival strategies to adapt to the thermal environment. The heat shock proteins (Hsps) play a crucial role in managing protein concentration, conformation, and subcellular location, especially when numerous stresses such as high temperature are applied. Expectedly, the E412 genome had numerous homologs of genes coding for Hsps (Supplementary Table 2). The homologs of the *clp* family (*clpB, -C, -P, -S, -X*) belonged to the Hsp100 family and helped maintain protein homeostasis (Labreck et al., 2017). The homologs of *htpG* protein of the Hsp90 family were likely to be primarily involved in protecting the photosynthetic machinery from heat stress (Takeshi et al., 2010). The homologs belonging to the Hsp70 family, mainly including *dnaK* and *dnaJ*, were abundant in the E412 genome. However, *dnaK* and *dnaJ* proteins might have different functions, and only part of them was responsible for thermotolerance (Duppre et al., 2011). Besides, a homolog of *grpE*, as a cofactor of the Hsp70 family, may help to prevent the aggregation of heat-denatured proteins (Schneider, 2011). As for the Hsp60 family, two distinct homologs of *groEL* genes, also referred to as the *groE* chaperone machinery, were identified in the E412 genome, and only one of them formed *groESL* operon with *groES*. This composition was similar to that of *T. sichuanensis* A183, but distinguished from that of *Gloeobacter* PCC 7421, the genome of which contains two *groESL* operons. Thus, we speculated that one *groESL* operon of the two strains lost the *groES* during the evolutionary process.

### Photosynthesis

The E412 genome comprised the homologs of complete sets of genes coding for both photosystem I (14 genes; some with additional homologs) and photosystem II (22 genes; some with additional homologs) (Supplementary Table 2). The genome also had homologs of genes coding for phycobilisome proteins, allophycocyanin, phycocyanin, and phycoerythrin. The genes related to phycoerythrin (blue-light absorbing) might favor the strain to acclimate to low light intensity and oligotrophic environment (Tang et al., 2019). These genomic features strongly support the described experimental evidence for the chromatic adaptation capacity of this strain.

Surprisingly, the E412 genome harbored no homologs of genes encoding photoprotective proteins, such as flavodiiron proteins and orange carotenoid proteins. This result suggested that strain E412 probably copes with photodamage by alternative acclimation mechanisms in light of high altitude and high-light exposure in the environment of its origin. Interestingly, flavodoxin gene (*fldA*) and iron stress-inducible proteins (*isiA*) were found in the E412 genome. The presence of the two genes indicated that under iron-deficient conditions, strain E412 might use flavodoxins to substitute ferredoxin in electron transfer and to increase the light-absorbing efficiency of PSI in the form of PSI-*isiA*-flavodoxin supercomplex (Cao et al., 2020).

Essential genes required for the Carbon-dioxide Concentrating Mechanism (CCM) were present in the E412 genome (Supplementary Table 2). The uptake of gaseous CO_2_ systems in cyanobacteria relied on NADPH dehydrogenase (NDH-1) complexes. The E412 genome included two NDH-1 complexes: a low-CO_2_ inducible high-affinity NDH-1_3_ complex encoded by *ndhD3*, *ndhF3*, and *cupA* (*chpY*) genes, and a constitutive low-affinity NDH-1_4_ complex encoded by *ndhD4*, *ndhF4*, and *cupB* (*chpX*) genes. In addition, our previous study indicated that strain E412 can utilize bicarbonate as a one of the sources of inorganic carbon (Tang et al., 2018a). The genome analysis verified the experimental results through the presence of homologs of low affinity, high flux, Na^+^-dependent bicarbonate transporters (*bicA1* and *bicA2*) and several ABC-type bicarbonate transporters. Moreover, the genome had 21 *Hat*/*HatR* gene homologs encoding High-affinity carbon uptake protein. A similar abundance of *Hat*/*HatR* gene homologs was noticed in many strains, e.g., *Acaryochloris marina* and *Lyngbya aestuarii* BL J. On the contrary, strains like *Synechococcus* sp. WH8102 and *Synechocystis* sp. PCC 6,803 contain none. The prosperity of the *Hat*/*HatR* gene in E412 may be related to the mat habit of its origin, where diffusion becomes the primary transport mechanism for substrates and products of metabolism and can cause diffusion limitations to photosynthesis (Kothari et al., 2013).

### Nitrogen Metabolism

Cyanobacteria can utilize various organic and inorganic nitrogen sources, a feature vital for survival in oligotrophic environments (Esteves-Ferreira et al., 2018). The E412 genome had the homologs of genes encoding nitrogenases, including *nifB*, *nifE*, *nifH*, *nifN*, *nifW*, and *nifX* (Supplementary Table S2). The genomic composition suggested that strain E412 was a nitrogen-fixing cyanobacterium, further verified by nitrogenase activity test (Supplementary Figure 6). Interestingly, strain E412 hosted a homolog of the gene *hetR*, the master regulatory gene involved in heterocyst formation, although it was not found to develop heterocysts under conditions tested.

The experimental result showed the utilization of both nitrate (3 mM) and nitrite (6 mM) by strain E412 for active growth. Tracing back to the genetic basis, the E412 genome possessed the homologs of genes encoding ABC-type nitrate transport system, which were clustered and oriented in the same direction with two essential nitrogen-related genes encoding ferredoxin-nitrite reductase (*nir*) and ferredoxin-nitrate reductase (*nar*), respectively (Supplementary Table 2). This result was in accordance with many freshwater cyanobacterial strains (Maeda et al., 2015). In addition, this strain had the homologs of an ammonium transporter and homologs of both glutamine synthetase and glutamine amidotransferase, which played the primary role of ammonium ion assimilation (Muro-Pastor et al., 2005). The homolog of the gene coding for *ntcB* was also present in this strain, which may function as a nitrogen control system to efficiently utilize intracellular resources in adaptation to changing nitrogen availability in the natural environment (Aichi and Omata, 1997).

## Conclusion

In the present study, we have morphologically, physiologically, phylogenetically, and taxogenomically characterized a novel thermophilic cyanobacterium, strain E412 isolated from Lotus Lake hot spring situated in Ganzi prefecture, China. Results of polyphasic analysis suggested that strain E412 was a novel genus within the family Leptolyngbyaceae. Consequently, we have proposed a new genus *Leptothermofonsia sichuanensis Daroch, Tang and Shah et al gen. sp. nov.* as a best described to date representative of this taxon and proposed its delineation. Phylogenomic inference, average nucleotide/amino-acid identity, and the POCP between genomes supported the delineation of genus *Leptothermofonsia*. Furthermore, the obtained complete genome of *Leptothermofonsia* E412 facilitated the elucidation of genetic basis regarding genes related to thermotolerance, photosynthesis, and nitrogen metabolism. Furthermore, experiments regarding chromatic adaptation capacity and utilization of sodium bicarbonate, sulfur, and nitrogen compounds confirmed these physiological characteristics as indicated by the genome sequence. Additionally, approximately a quarter of the annotated protein-coding genes in the E412 genome could be acquired through HGT and may impact genome expansion and habitat adaptation. Overall, the complete genome of strain E412 provides the first insight into the genomic feature of the genus *Leptothermofonsia* and lays the foundations for future global ecogenomic and geogenomic studies.

Taxonomic Treatment and Description of *Leptothermofonsia sichuanensis* Daroch, Tang, and Shah et al. gen. nov

Phylum: Cyanobacteria

Order: Synechococcales

Family: Leptolyngbyaceae

*Description*: Cells brownish colored; filamentous; coiled or tangled filaments; sheath colorless, very thin, not lamellate; trichome slightly constricted and composed of cylindrical, elongated cells; narrow trichome; apical cells are round; cells longer than broad; no false branching (Figure 4A). Trichomes unbranched and composed of cylindrical, elongated cells, 1.2–1.8 μm in length and 0.8–1.1 μm in width. Cross-walls of the cells contained constrictions (Figures 4B,C). Centripetal invagination of the cell wall divided individual cells, and intracellular connections between vegetative cells were not present (Figure 4C). Five to six thylakoids in the parietal arrangement were located parallel at the cells’ inner periphery. Sheath, septum, phycobilisome, carboxysomes, cyanophycin granule, lipid droplets, and polyphosphate bodies were all present in the cytoplasm (Figures 4C,D).

Type strain: is E412 (= FACHB-2490).

*Type species*: *Leptothermofonsia sichuanensis Daroch, Tang, and Shah et al. gen*. *nov*. (see below).

*Etymology*: “Lepto” exhibiting morphology typical to the members of Leptolynbyaceae family, “thermo” similar to thermophilic (high temperature tolerant), “fonsia,” genus epithet derived from the Latin word *fons* meaning spring, since both representative of the genus to date, i.e., E412 and Greenland 10, are hot spring isolates; “sichuanensis” species epithet derives from the name of collection province.

*Type locality*: Thermal spring, Lotus Lake in Ganzi Prefecture of Sichuan Province, China.

Ecology of type locality: the sample occurred as a macroscopic brown-green submerged in the pond. Sample collection was done on 12.05.2016, with the humidity close to 71%. At the time of collection, the air temperature was 15°C, and the light intensity was around 1,000 lux. The pH of the spring was 6.32, and the concentration of total dissolved solids was 447 mmol L^–1^.

*Habitat*: Thermal springs in Ganzi Prefecture of Sichuan Province, China (30°05′14″ N, 101°56′55″ E). This species (strain E412) is capable of bicarbonate assimilation (Tang et al., 2018a). The genome has the homologs of genes encoding nitrogenases, including *nifB*, *nifE*, *nifH*, *nifN*, *nifW*, and *nifX* (Supplementary Table 2). The strain was experimentally confirmed as nitrogen-fixing. Interestingly, this strain hosted a homolog of the gene *hetR*, the master regulatory gene involved in the formation of heterocyst, although it does not develop heterocyst. Strain shows the capacity of chromatic adaptation by modifying the content of phycobilisomes depending on light conditions.

*Holotype here designated*: the culture of *Leptothermofonsia sichuanensis Daroch, Tang and Shah et al.* gen. sp. *nov*. was initially denoted and deposited in Peking University Algae Collection as PKUAC-SCTE412 has also been deposited in the Freshwater Algae Culture Collection at the Institute of Hydrobiology (FACHB-collection) with accession number FACHB-2490 as *Leptolyngbya* species after identification and authentication based on the full-length sequencing of the 16S rRNA gene along with folding of the secondary structures of the 16S-23S ITS region. After proper identification and authentication, the culture is maintained in the FACHB under the accession number FACHB-2490.

## Data Availability Statement

The complete genome sequence reported in this study has been deposited in GenBank with an accession number CP072600.

## Author Contributions

JT: conceptualization, methodology, validation, formal analysis, investigation, data curation, writing—original draft, writing—review and editing, visualization, supervision, project administration, and funding acquisition. MS: investigation, methodology, validation, and writing—original draft. DY: formal analysis, software, and data curation. YJ: formal analysis, software, data curation, and writing—review and editing. LD, MW, and MMW: methodology, software, and data analysis. KZ: software and data curation. LL: formal analysis, investigation, data curation, and writing—original draft. ML: formal analysis, investigation, and data curation. KW: conceptualization, methodology, writing—review and editing. MD: conceptualization, methodology, resources, data curation, writing—original draft, writing-review and editing, supervision, project administration, and funding acquisition. All authors contributed to the article and approved the submitted version.

## Conflict of Interest

The authors declare that the research was conducted in the absence of any commercial or financial relationships that could be construed as a potential conflict of interest.

## Publisher’s Note

All claims expressed in this article are solely those of the authors and do not necessarily represent those of their affiliated organizations, or those of the publisher, the editors and the reviewers. Any product that may be evaluated in this article, or claim that may be made by its manufacturer, is not guaranteed or endorsed by the publisher.

## Footnotes

1
https://www.genome.jp/kegg/ko.html

2
http://enve-omics.ce.gatech.edu/g-matrix/
