# Supplementary material for: Polyphasic Identification and Genomic Insights of Leptothermofonsia sichuanensis gen. sp. nov., a Novel Thermophilic Cyanobacteria Within Leptolyngbyaceae
Source: Front Microbiol. 2022 Mar 28;13:765105. doi: 10.3389/fmicb.2022.765105 (PMC8997340; doi:10.3389/fmicb.2022.765105)
Supplement: Supplementary file 1 [file Data_Sheet_1.PDF]

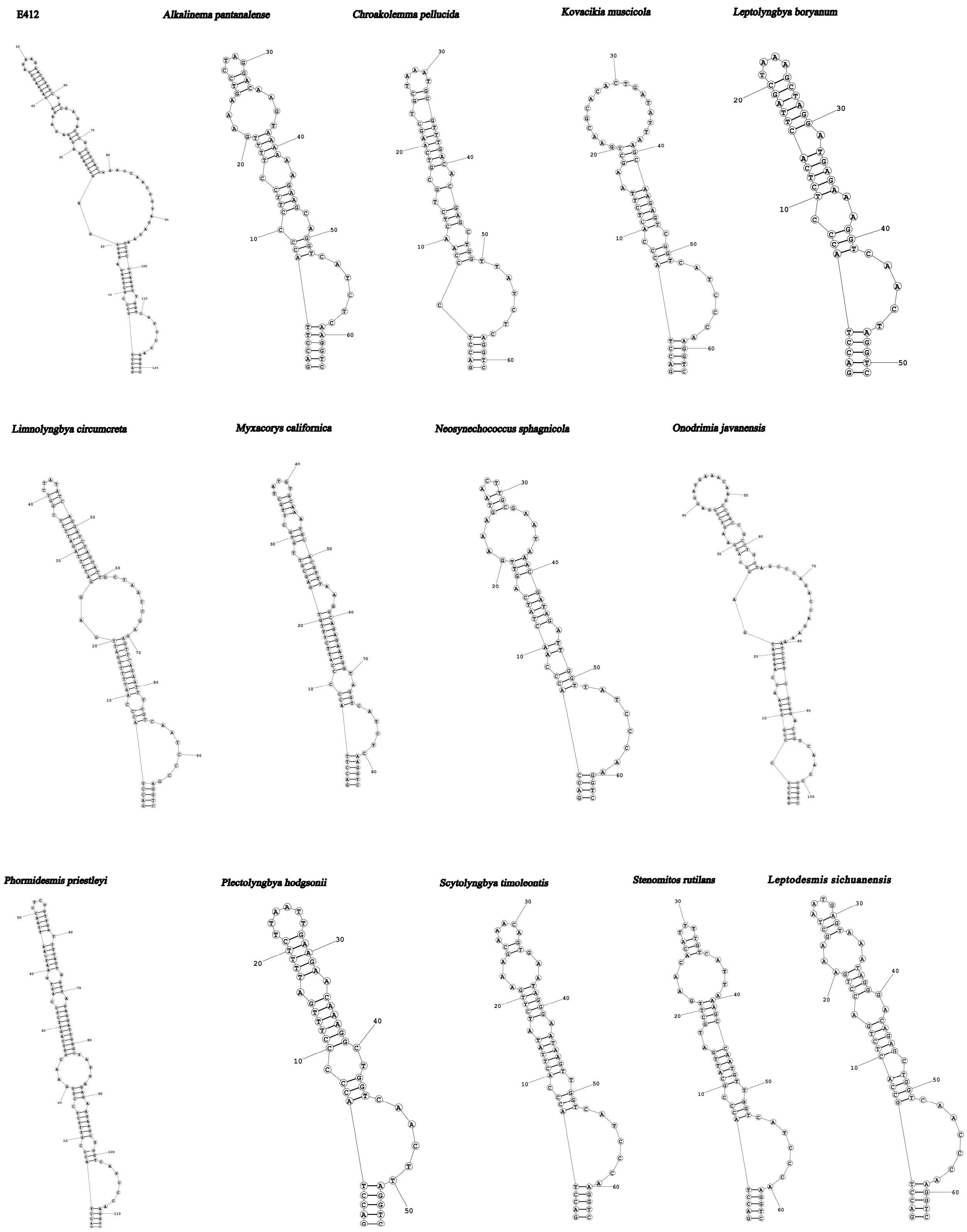

Supplementary Figure 1 Predicted secondary structures of D1-D1' region within 16S-23S ITS of Leptolyngbyaceae strains.

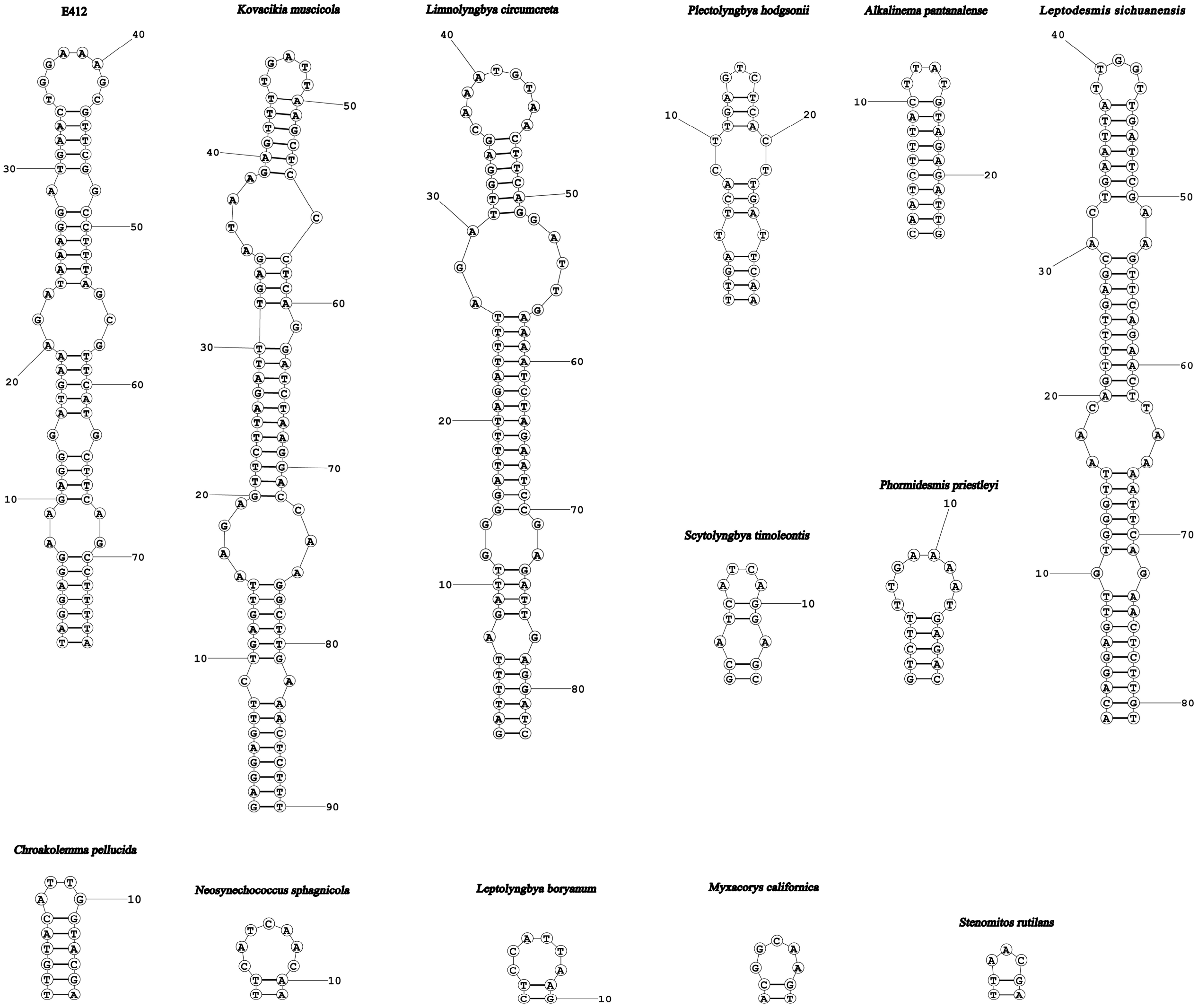

Supplementary Figure 2 Predicted secondary structures of V2 region within 16S-23S ITS of Leptolyngbyaceae strains.

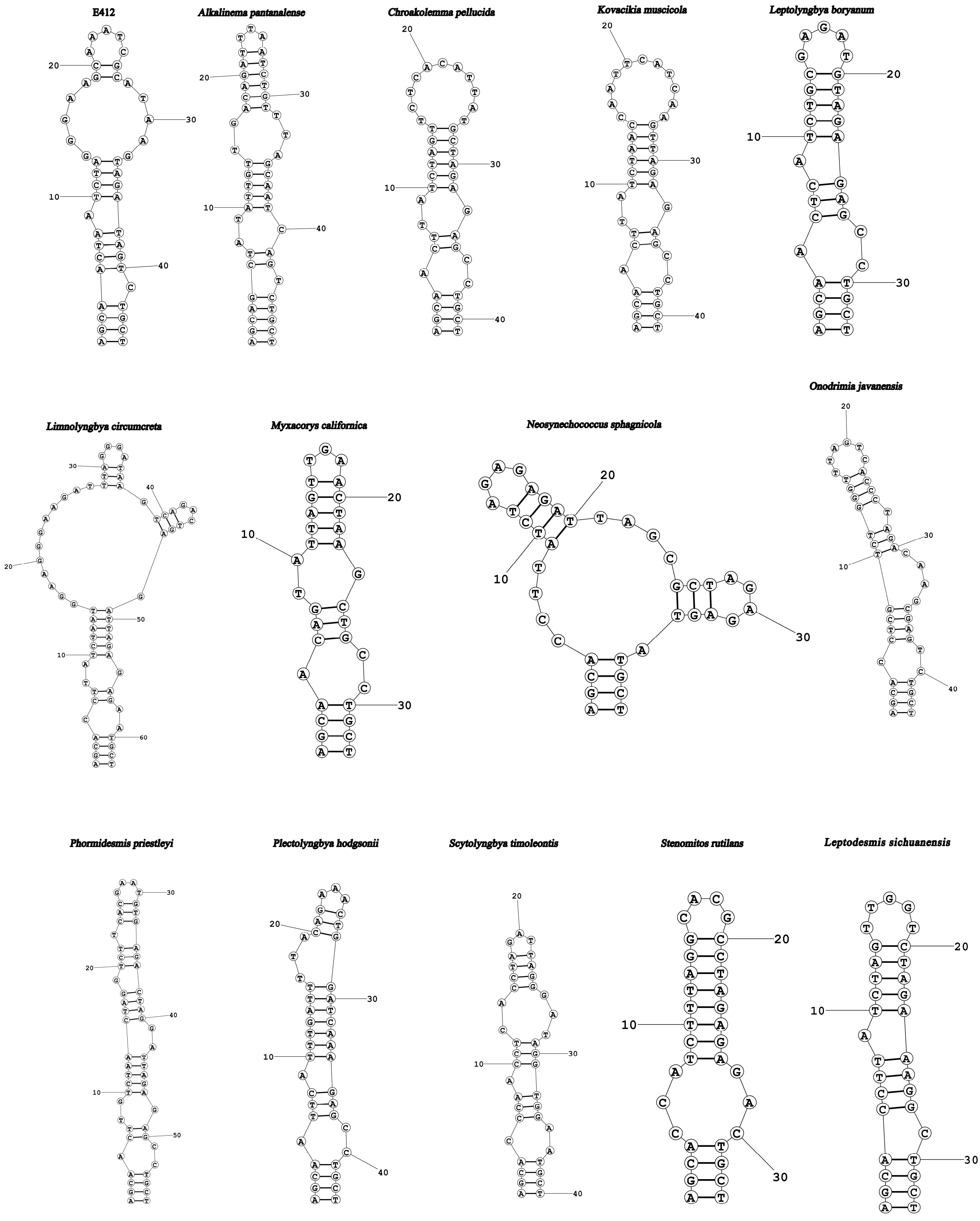

Supplementary Figure 3 Predicted secondary structures of boxB region within 16S-23S ITS of Leptolyngbyaceae strains.

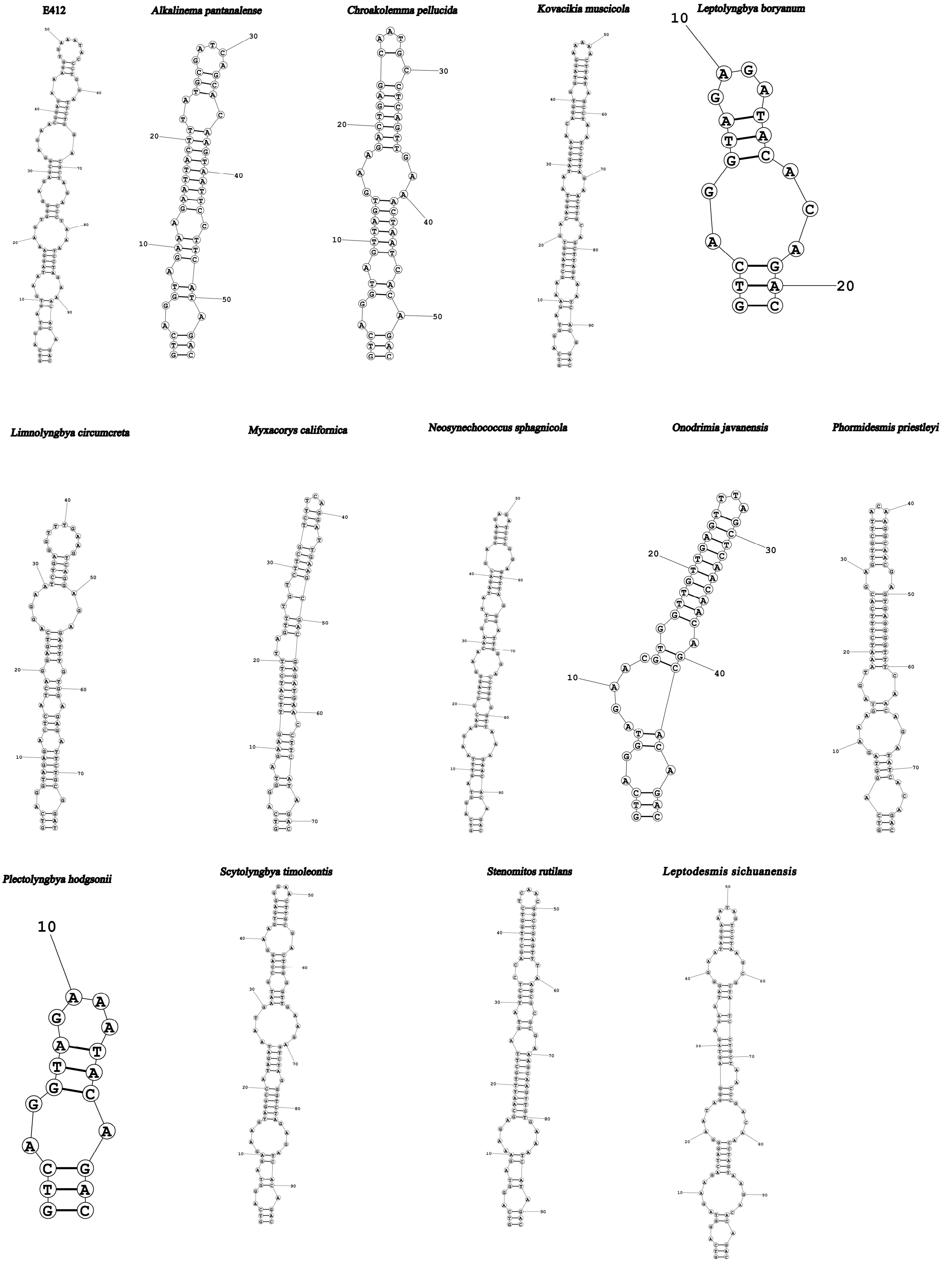

Supplementary Figure 4 Predicted secondary structures of V3 region within 16S-23S ITS of Leptolyngbyaceae strains.

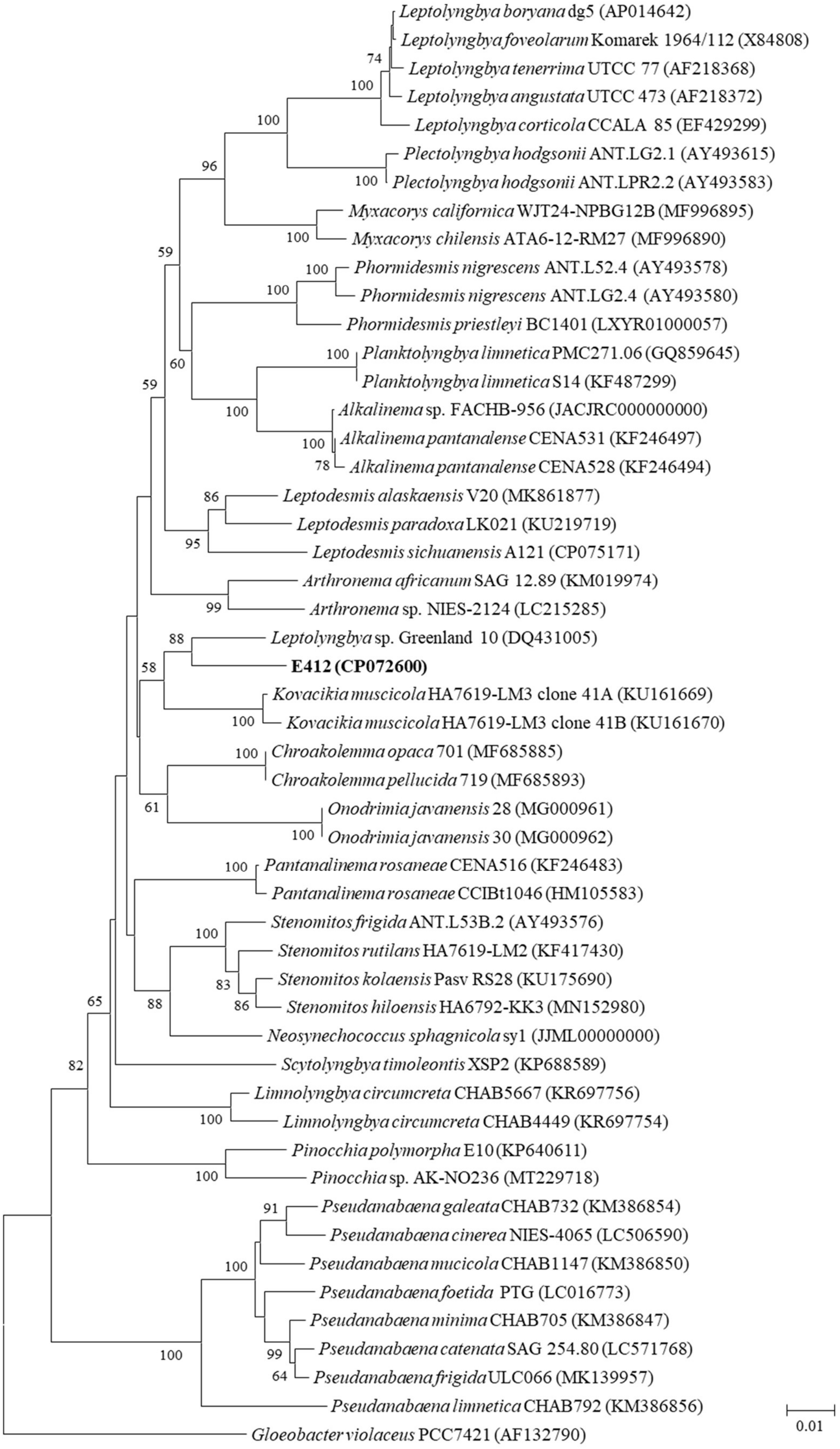

Supplementary Figure 5 Neighbor-joining phylogenetic tree of 16S rRNA gene sequences. Strain no. in bold represent the strains identified in this study.

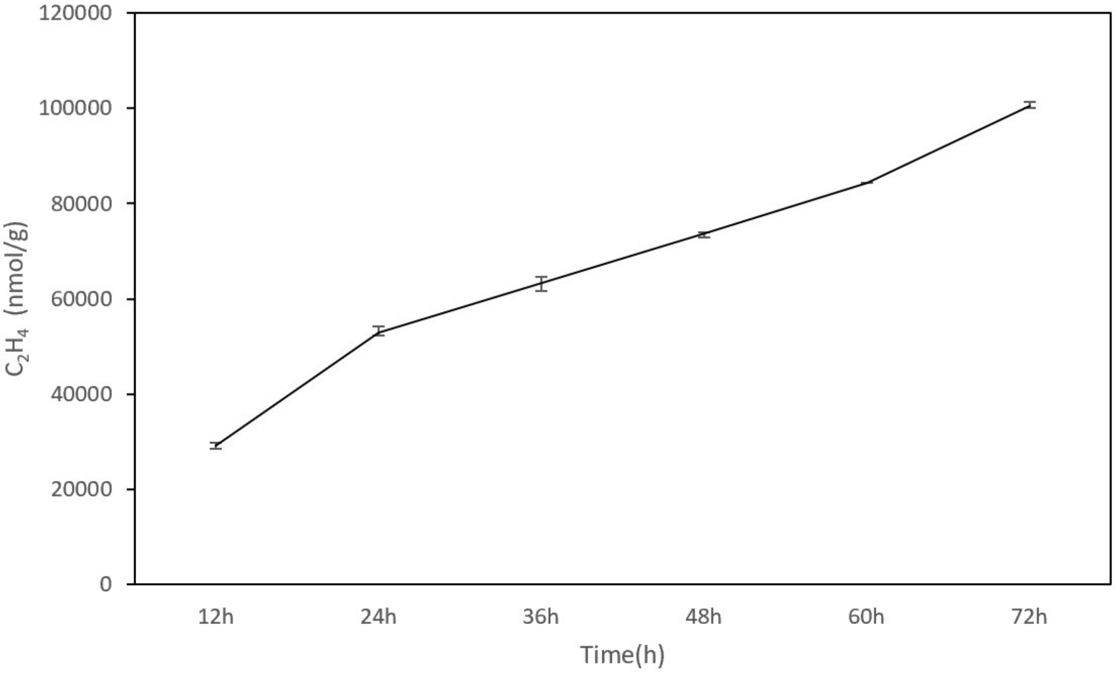

Supplementary Figure 6 Nitrogenase activity of cell suspensions of *Leptothermofonsia sichuanensis* gen. nov. E412 represented as a acetylene reduction proxy assay. Assay presents nmol/gcells of ethylene released from the strain grown in nitrogen-free BG-11 medium during 72 h, 45℃, 30 μmol m^{-2} s^{-1} gas composition $Ar/N_2/CO_2$ = 90/9/1 [v/v/v %].

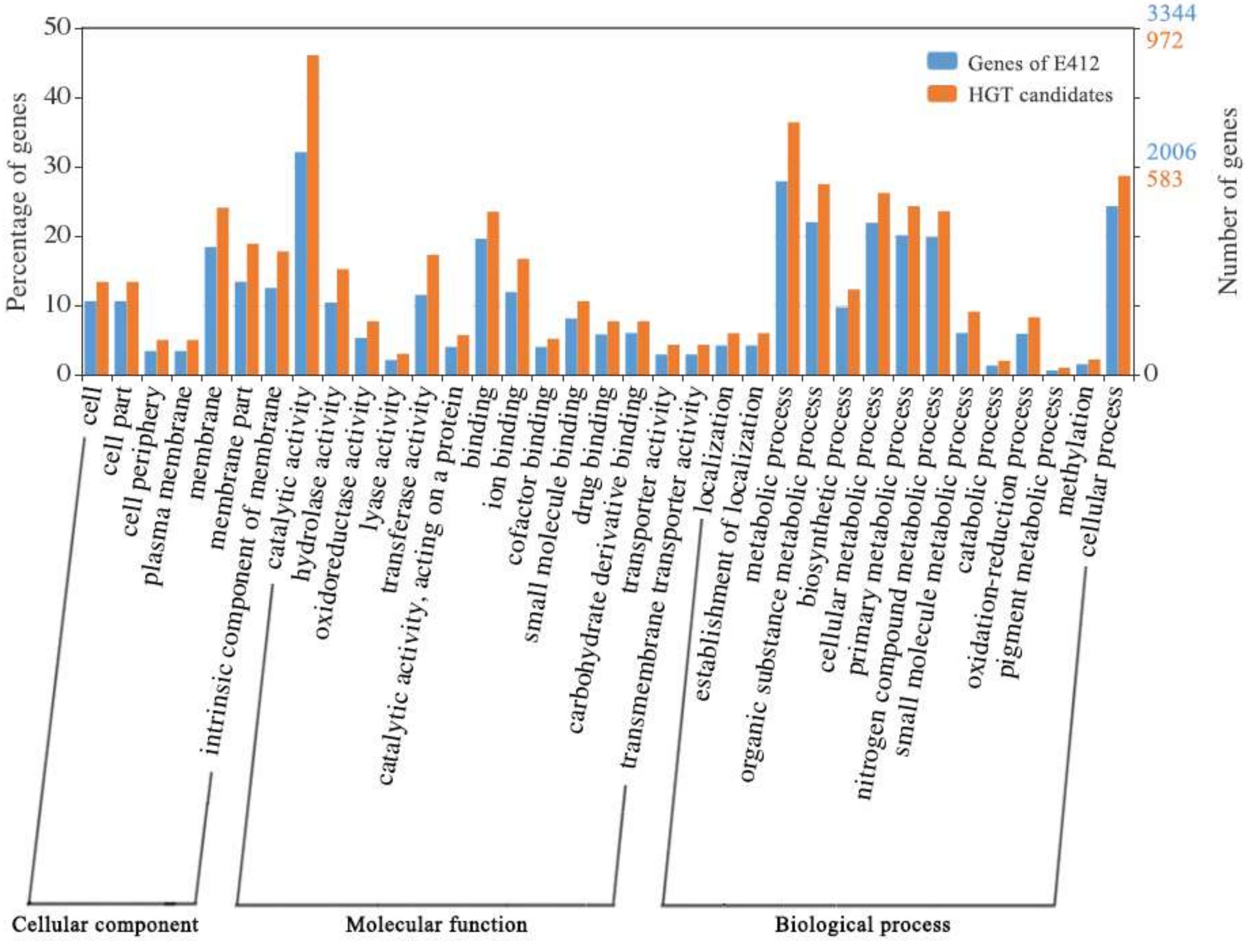

Supplementary Figure 7 Gene ontology and functional classification of genes identified in E412 genome.

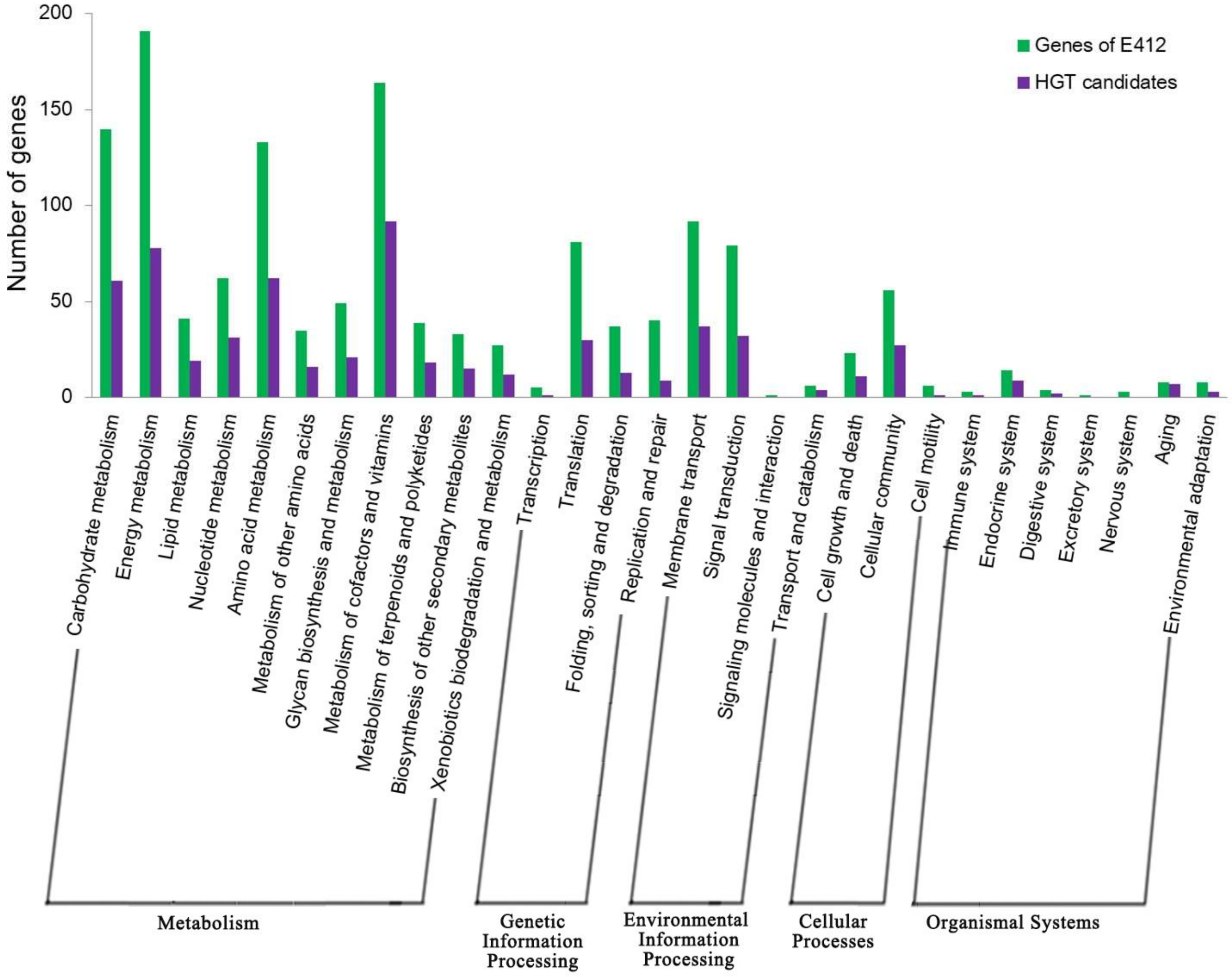

Supplementary Figure 8 Fuctional classification of protein-coding genes identified in E412 genome based on KEGG orthology.
